# Microstructure and Shear Strength of SiC Ceramics Diffusion Bonded with Ti Foils by Spark Plasma Sintering

**DOI:** 10.3390/ma18081725

**Published:** 2025-04-09

**Authors:** Weiqin Hu, You Xie, Xinru Xu, Tengfei Yang, Haocheng Liu, Rui Tu, Ruixuan Tan

**Affiliations:** 1 School of Materials Science and Engineering, Hunan University, Changsha 410082, China; weiqin_hu@163.com (W.H.); xieyou202202@163.com (Y.X.); 15252576325@163.com (X.X.); 2 Research Institute, State Power Investment Corp., Ltd., Beijing 102209, China; liuhaocheng@spic.com.cn (H.L.); turui4y@163.com (R.T.)

**Keywords:** diffusion bonded, SiC ceramics, spark plasma sintering, shear strength

## Abstract

This study systematically investigated the diffusion bonding of SiC ceramics using Ti interlayers via spark plasma sintering. The effects of holding time (5 min, 10 min, 20 min), joining temperature (1300 °C, 1400 °C, 1500 °C), and Ti foil thickness (5 μm, 10 μm, 20 μm) on joint microstructure and shear strength were characterized through SEM, TEM, XRD, and EDS. The results revealed that the joining temperature was the key factor influencing joint performance, while the holding time and Ti foil thickness had relatively minor effects. When joined at 1300 °C, the primary reaction products in the interlayer consisted of TSC, TiC, and Ti_5_Si_3_ phases. Prolonged holding times improved the shear strength, but inhomogeneous phase distribution adversely affected the stability of mechanical properties. In joints with thinner Ti interlayers (5 μm and 10 μm), Si-rich phases tend to aggregate in the central region, leading to stress concentration and reduced joint strength. As the joining temperature increased to 1400 °C and 1500 °C, the Ti_5_Si_3_ phase was gradually consumed, and the interlayer primarily contained TSC and TiC phases, leading to a significant enhancement in shear strength, with a maximum value of 127.67 MPa achieved at 1400 °C. These findings provide important experimental evidence and theoretical support for optimizing the joining process of SiC ceramics.

## 1. Introduction

Silicon carbide (SiC) ceramics and their composites are regarded as promising materials to replace zirconium (Zr)-based alloys as the fuel cladding materials for light water reactors (LWRs) in the field of nuclear power due to their combination of outstanding properties, such as excellent high-temperature capability, remarkable chemical stability, a relatively low neutron absorption cross-section, and better resistance to irradiation damage [1,2]. Since the occurrence of the Fukushima nuclear accident in Japan in 2011, the demand for researching and developing SiC-based composites as novel accident-tolerant fuel (ATF) cladding materials has become increasingly urgent [3]. The airtightness of the cladding materials for nuclear reactors is of crucial importance. Even tiny cracks or pores may lead to the leakage of fission gases and nuclear fuel. In order to isolate radioactive substances from the external coolant, the cladding materials must be sealed with end cups by means of connection techniques for using in the nuclear reactors [4]. On the other hand, extremely high requirements are imposed on the quality and integrity of the cladding materials. Due to the limitations of forming techniques and equipment for SiC ceramics, the preparation of large-sized and complex-shaped components has always been a challenge. Connecting simple or small parts into large-sized components with complex shapes is currently a relatively effective method [5]. However, because of the existence of strong Si–C covalent bonds and a low self-diffusion coefficient within SiC itself, it is extremely difficult to effectively join it with end cups using welding techniques similar to those used for metals and alloys [6]. Therefore, the development of a joining technology that can enable an effective and high-strength connection between the cladding tube and the end cup is a crucial issue regarding whether SiC ceramics and SiC composites can be applied as ATF in the advanced nuclear reactors [7,8].

Till now, numerous promising joining technologies for SiC/SiC and SiC/other different materials have already been developed, such as active metal brazing-based joining [9,10], reaction bonding joining [11,12], preceramic polymer joining [13], transient eutectic phase joining [14], solid-state diffusion bonding [15,16,17], and MAX phase joining [18]. These joining technologies can be realized using various processes such as hot pressing or conventional sintering [19,20], and selective laser beam sintering [21]. However, in order to achieve high-strength joining, most of the sintering processes usually rely on high temperature, high pressure, and relatively long times for joining. For examples, Fitriani et al. [16] used thin Ti_3_AlC_3_ and Ti_3_SiC_2_ MAX phase fillers to join SiC ceramics at 1700 and 1900 °C for 5 or 8 h under a pressure of 3.5 MPa, and the joining strength reached 300 MPa and 230 MPa, respectively. Wu et al. [22] used CeO_2_-Al_2_O_3_ as the interlayer material to join SiC ceramics through the transient eutectic phase. When the joining temperature was 1700 °C, the shear strength of the SiC joint was 163.9 MPa. However, when the joining temperature decreased to 1500 °C, the shear strength of the joint also dropped to 57.4 MPa. Whether it is high temperature, high pressure, or prolonged heating, all of these factors can have an adverse impact on the performance of the cladding materials. For the cladding materials with thin-walled structures, it is not advisable to apply relatively high pressure during the joining process [23]. Furthermore, considering that the length of ATF of SiC and SiC composites would reach 4 m and the thermal stabilities of nuclear fuels, these joining approaches probably are not suitable for the applications of SiC and SiC composites fuel cladding in the advanced nuclear reactors.

Spark plasma sintering (SPS, also known as electric current field-assisted sintering technology, FAST), as an advanced ceramic joining technology, possesses the advantages of rapid heating rate, short sintering time, relatively low sintering temperature, and the ability to effectively control the microstructures of materials, and it has been successfully applied in nuclear fuel manufacturing [24]. For example, within a holding time of 10 min, SPS has successfully connected SiC with Zr alloys, ZrB_2_-SiC, and MAX phase at the corresponding joining temperatures of 1450 °C, 1600 °C, and 1250 °C, respectively [25,26,27]. Due to the characteristic of local heating, the SPS sintering process accelerates the atomic diffusion rate at the interfaces, making it a highly promising ceramic joining technology for SiC and SiC composites [28].

Since the joint interlayer is generally believed as the weakest part of the entire fuel cladding component in extremely harsh environments with high temperature, high pressure, and high irradiation, the microstructures, chemical compositions, and strength of the joint interlayer have been widely studied [29]. Ti is the most widely used starting material of the intermediate layer for the joining of SiC for advanced nuclear systems due to its reactivity with the SiC, and the high strength, great corrosion resistance, and irradiation resistance of the intermediate layer formed by the reaction of Ti and SiC [30]. The active metal Ti is an excellent candidate for one of the intermediate layers for joining SiC. At high temperatures, it reacts with SiC to form the ternary MAX phase Ti_3_SiC_2_ (TSC). TSC combines the outstanding properties of both metals and ceramics and effectively enhances the interfacial bonding strength [31,32,33]. Gottselig et al. [34] conducted joining experiments on Ti and SiC under the conditions of 1450 °C and 5–30 MPa. Their research demonstrated that the formation of TSC led to high-strength joining (with a measured flexural strength of 286 MPa). Rizzo et al. [35] joined C/SiC composites with Ti foils of different thicknesses (30 μm and 130 μm). The results showed that the main phases in the intermediate layer were the TiC phase and the TSC phase. For the thicker Ti intermediate layer, a Si-rich phase (possibly a Ti-Si metallic phase) was also detected. Grasso et al. [36] achieved the joining of a 30 μm Ti foil with β-SiC at a joining temperature of 1700 °C and obtained SiC joints with a flexural strength of 126 ± 16 MPa. The main phase detected in the intermediate layer was TiC. However, the excessively high temperature (>1500 °C) that causes the decomposition of TSC and the evaporation of Si, as well as the use of a relatively thick joining layer (>30 μm) which makes the residual Ti continuously react to form the Ti-Si metallic phase, are both unfavorable for applications in nuclear environments [37]. On the other hand, the evolutions of microstructures, chemical compositions, and strengths of the joint interlayer with the temperature and time of SPS are still not clear, which limits the further optimization of joining parameters and the enhancement of joint strength.

In this study, a series of solid-state diffusion joining experiments with SPS were designed and carried out to reveal the evolutions of microstructures and chemical compositions of joint interlayers with the time and temperature of SPS. Pure Ti foils were employed as the starting material of the intermediate layer for diffusion bonding of SiC ceramics. The shear strengths of joint interlayers were also measured and compared. Transmission electron microscopy (TEM) and scanning electron microscope (SEM) characterizations reveal that when SiC and Ti are joined at 1300 °C, the reaction products in the interlayer are independent of both the holding time and the thickness of the Ti foil. At this temperature, the interlayer primarily consists of three phases: TSC, TiC, and Ti_5_Si_3_. While longer holding times can improve the shear strength of the joint, they also tend to cause inhomogeneity in the interlayer microstructure, leading to inconsistent shear strength in the fabricated joints. When a thinner Ti foil is used as the interlayer, it often results in the aggregation of Si-rich phases in the central region of the interlayer, creating stress concentration and consequently reducing the joint strength. As the temperature increases, the reaction between Ti and SiC becomes more complete, resulting in a more uniform microstructure and higher shear strength. These experiment results would provide important evidence for understanding the reaction and atomic diffusion processes during SiC joining with Ti as the intermediate layer using SPS, and further optimizing the joining parameters to improve the joint strength.

## 2. Materials and Methods

### 2.1. Starting Materials

The pressure-less sintered SiC plates (with a density of 3.12 g·cm^−3^ and a purity of 99%) were purchased from Fuzhou Pengkun Optoelectronic Technology Co., Ltd., Fuzhou, China, with a size of 50 mm × 50 mm × 8 mm. The high-purity Ti foils (with a purity of 99.99%) were used as the joining interlayer, which were purchased from Shenzhen Haiyuan Scientific Research Metals. The thicknesses of the Ti foils are 5 μm, 10 μm, and 20 μm, respectively.

### 2.2. Process of Joining

**Figure 1:**
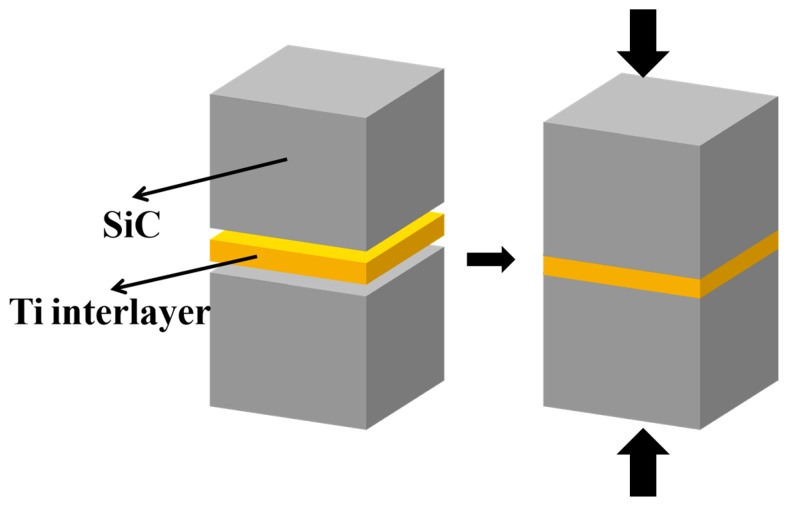
The SiC-Ti-Ti sandwich structure.

Before joining, the SiC plates were machined into 10 mm × 10 mm × 8 mm cubes. The joining surfaces of the two SiC cubes were sanded and polished using diamond sandpapers with mesh numbers ranging from low to high, namely 1200, 2000, 3000, 4000, 8000, and 9000, respectively, as well as diamond-polishing slurry with a particle size of 0.1 μm; all the SiC cubes were ultrasonically cleaned with ethanol for 30 min. The Ti foils were placed between two SiC cubes and fixed to form a SiC-Ti-SiC sandwich structure (Figure 1). The SiC-Ti-SiC samples were loaded into a graphite die, and then placed into a SPS furnace (Model SI: LABOX-325R). The joining temperature of the equipment was controlled by a pyrometer placed on the top, which monitored the temperature of the joining process. All the samples were heated in vacuum under pulsed current (range from 800 A to 1200 A), with both the heating rate and the cooling rate being 100 °C·min^−1^, and the applied uniaxial pressure was 30 MPa. The microstructure and shear strength of the joint interlayers prepared by SPS under different temperatures (1300 °C, 1400 °C, and 1500 °C), dwell times (5 min, 10 min, and 20 min) and thicknesses of Ti foils (5 μm, 10 μm, and 20 μm) were investigated and compared.

### 2.3. Shear Strength Measurements

**Figure 2:**
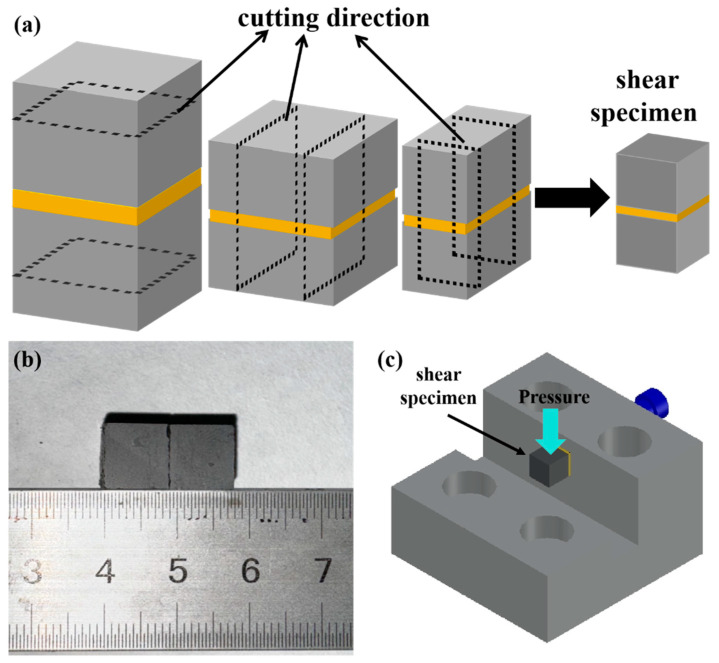
(**a**) The cutting direction for shear specimen; (**b**) SiC-Ti-SiC joint; (**c**) shear strength testing device.

After joining, all the samples were cut into 10 mm × 5 mm × 5 mm rectangular bars by a diamond wire cutting machine (STX-202AQ, purchased from Shenyang Kejing Automation Equipment Co., Ltd., Shengyang, China.), and then the surfaces were polished. Figure 2a shows the cutting direction for shear strength samples. The shear strengths were measured using a self-made device in the laboratory (Figure 2c), and the tests were performed on an AGX-V 50 kN electronic-tensile testing machine. The load speed for all measurements was set as 0.5 mm·min^−1^. The shear strength can be calculated using the following equation (Equation (1)):

*τ* = F/A (1)

where F was the applied load in N, A is the force-bearing area of each sample in mm^2^. For each joining condition, at least two specimens were tested to ensure data reliability while maintaining experimental efficiency. The resulting data were analyzed in terms of average values, peak values, and corresponding standard deviations.

### 2.4. Microstructural Characterizations

The microstructures and chemical compositions of joint interlayers were observed and analyzed using a scanning electron microscope (SEM, Zeiss Sigma 300 VP, Carl Zeiss AG, Jena, Germany) equipped with an energy dispersive spectroscopy (EDS) system and a spherical aberration-corrected transmission electron microscope (TEM, Themis Z (3.2), Thermo Fisher Scientific, Brno, Czech Republic). All the foils used for TEM observations were polished into circular thin sheets with a diameter of 3 mm and a thickness of about 60 μm from the cross-sectional samples by using diamond sandpapers. Subsequently, the samples were finally thinned by an ion milling apparatus (Fischione 1051, Fischione Instruments, Export, PA, USA) to form the thin regions that could be observed by TEM. Phase analysis of the fracture surfaces of the joints was conducted by XRD (MiniFlex600, Rigaku Corporation, Tokyo, Japan). The XRD test samples were prepared by grinding layer by layer, as the fracture locations of some samples occurred within the matrix.

## 3. Results and Discussion

### 3.1. Microstructure and Phase Composition of SiC-Ti-SiC Joints Under Different Dwell Times

**Figure 3:**
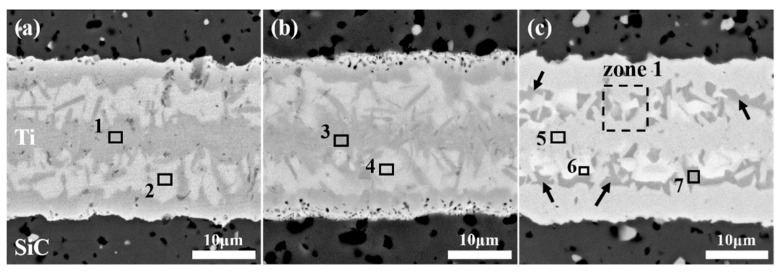
Microstructures of the polished cross-sections of joints under different holding times: (**a**) 5 min; (**b**) 10 min; (**c**) 20 min.

**Figure 4:**
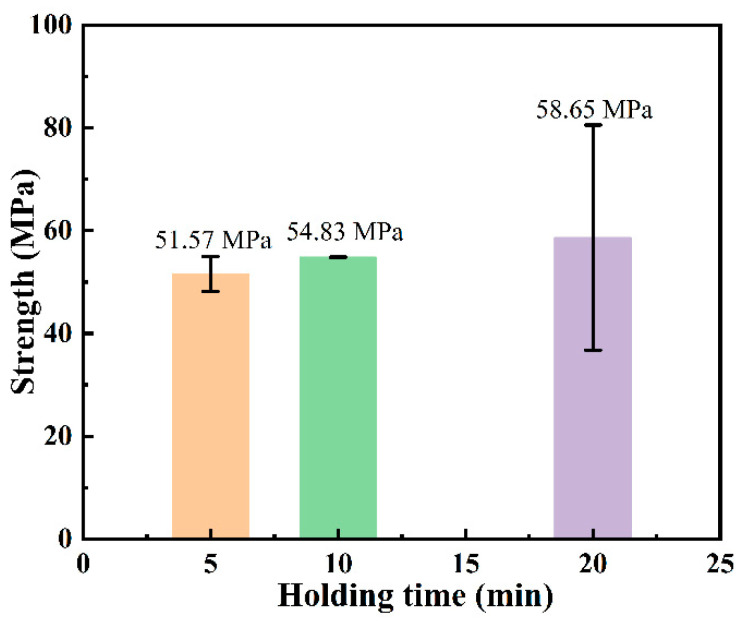
Shear strength of joints under different holding times.

Figure 3a–c show the polished cross-section microstructures of SiC ceramics joined with a 20 μm Ti foil at a temperature of 1300 °C under different holding times. The holding times are 5 min, 10 min, and 20 min, respectively, and the final thickness of the interlayer exceeds 26 μm in all cases. The results indicate that SiC ceramics can be successfully joined to the Ti foil by SPS even with a holding time of only 5 min. The interlayer region of all joints has a uniform thickness, which is closely and continuously connected to the SiC interface without cracks, and an obvious reaction layer can be observed. The interlayer regions of joints with different holding times always exhibit a similar microstructure, that is, the whole is a lamellar eutectic microstructure composed of a gray-contrast phase and a white-contrast phase. In the white-contrast phases on both sides, there are also gray-colored strip-shaped grains of various sizes interspersed. In the interlayer of the joint with a 20 min holding time, a decrease in the light-contrast phases on both sides is observed, and instead, a third-phase structure (brown-contrast phase, indicated by the black arrow in Figure 3c) appears, which is different from that of the other holding times. However, this situation does not exist continuously in the joint. Considering the local heating characteristic of SPS, there is a temperature gradient inside the sample during the joining process, resulting in different micro-morphologies. In addition, black pores are observed on the interlayer side at the interface with the SiC ceramic matrix, and agglomerated white-contrast phases fill the surface defects (pores or micro-cracks) of the matrix. It is worth noting that the pore size on the interlayer side is significantly smaller than that in the matrix, indicating that good densification has occurred in the interlayer region, and the degree of densification is higher than that of the SiC ceramic matrix. The highly dense interlayer provides a good foundation for high joint strength, which is also proven by the shear strength test results. As shown in Figure 4, the average shear strength of all SPS-joined SiC joints is above 50 MPa. With the increase in the holding time, the average shear strength of the joints gradually increases. The joint with a 20 min holding time has the highest shear strength (58.65 ± 21.89 MPa). When the holding time is reduced to 10 min, the shear strength drops to 54.83 ± 0.05 MPa. When the holding time is further shortened to 5 min, the shear strength finally decreases to 51.57 ± 3.41 MPa. The change in the holding time affects the degree of atomic diffusion and the progress of the reaction. The shorter the holding time, the less sufficient the atomic diffusion, and the fewer atoms participate in the reaction to generate the strengthening phase, resulting in a relatively lower joint strength. As the holding time is prolonged, atomic diffusion becomes more sufficient, and more strengthening phases such as TSC can be formed, effectively improving the joint strength. However, when the holding time is too long, local heating by SPS causes over-sintering in some regions of the joint interlayer, leading to problems such as abnormal grain growth and non-uniform microstructure (as shown by the black arrows in Figure 3c), which in turn affects the mechanical properties of the joint and causes a large deviation in strength. Therefore, the shear strengths of the two SiC joints prepared with a 20 min holding time have a large deviation. The joints prepared with a 10 min holding time have relatively high quality stability and will be regarded as the optimal holding time in subsequent studies.

**Figure 5:**
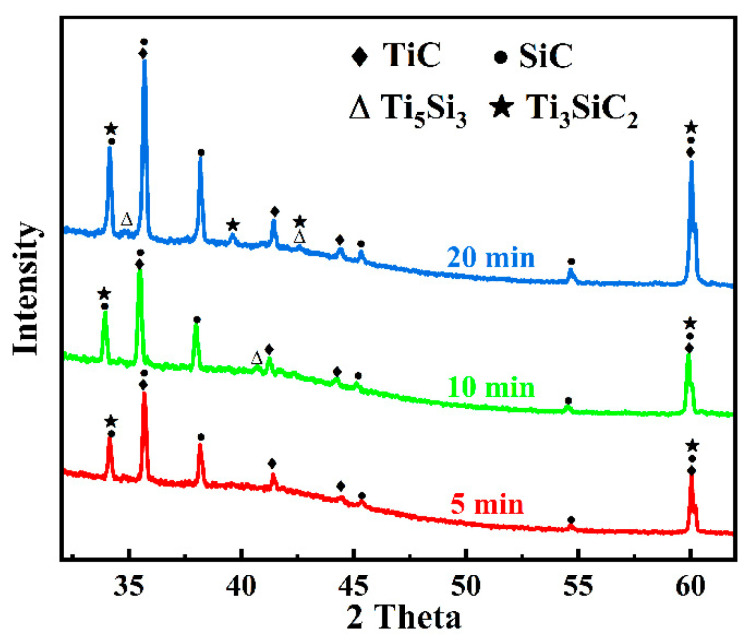
XRD of joints under different holding times.

The three different contrast phases in the joint interlayer region showed in Figure 3 are marked as points 1–7. Among them, points 1, 3, and 5 are gray-contrast phases, points 2, 4, and 6 are white-contrast phases, and point 7 is the third-phase structure. Table 1 presents the EDS analysis results of each point. Under different holding times, the interlayer region contains only three elements, Ti, S, and C. Although the white-contrast phase and the gray-contrast phase have similar chemical compositions, the white-contrast phase is obviously a Si-rich phase; the gray-contrast phase is a Ti + C-rich phase; the third-phase is also Si-rich, and the Ti:Si element ratio is close to 5:3. Combining with the isothermal equilibrium phase diagram of the Ti-Si-C ternary system [38], TiC phase, TSC phase, and Ti_5_Si_3_ metal phase may coexist in the joint region. After the shear strength test, the phase structure of the SiC joint interlayer was analyzed by XRD. As shown in Figure 5, the interlayer phases of the joint with a low holding time (5 min) are mainly TiC and TSC. When the holding time is extended to 10 min and 20 min, the interlayer phases detected are mainly TiC, TSC, and Ti_5_Si_3_. Zhang et al. [39] have studied and shown that metal Ti has a strong affinity for Si, C, and SiC at high temperatures and can undergo many reactions. Under the combined action of the SPS joining temperature and pressure, the diffusion rate of C atoms in the SiC substrate is higher than that of Si atoms. The C atoms reach the interlayer first and react with Ti to generate TiC. At the same time, some Si atoms that have already diffused into the interlayer will react with Ti and C to generate TSC. The following reactions explain the formation of the interlayer phase structure of the joint with a 5 min holding time [19,40]:

Ti + C → TiC (2)

Ti + Si + C → TSC (3)

In fact, the Ti_5_Si_3_ metal phase exists in the reaction products of joining SiC with Ti under long-term high-temperature conditions. Therefore, the Ti_5_Si_3_ phase was detected in the joint interlayers with holding times of 10 min and 20 min. For the joint with a 5 min holding time, due to the relatively short holding time, the content of Ti_5_Si_3_ generated by the reaction is relatively small, and it was not detected by XRD. With the increase in the holding time, the in situ-generated TiC will continue to react with the remaining Ti and Si to generate TSC. Therefore, more TSC phases were detected in the joint with a 20 min holding time, which is consistent with the previous report by Yang et al. [41]. The possible reactions are as follows:

Ti + Si → Ti_5_Si_3_ (4)

Ti + SiC → TiC + Ti_5_Si_3_ (5)

TiC + Ti + Si → TSC (6)

**Figure 6:**
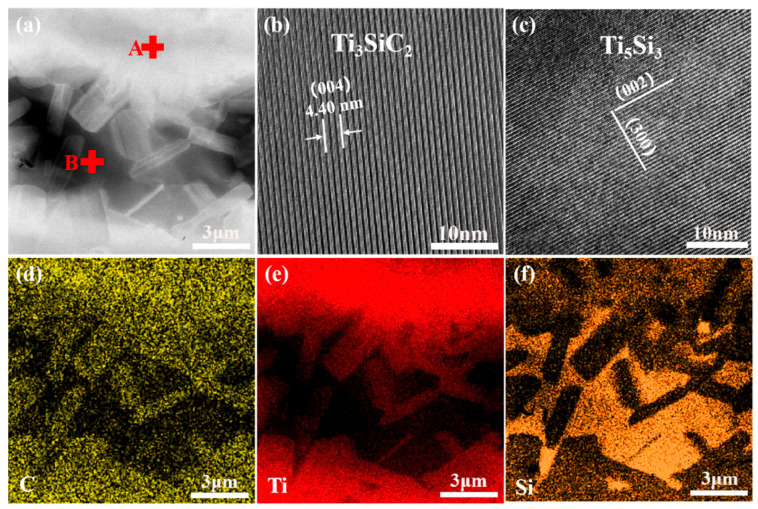
(**a**) TEM image of joint with 20 min; (**b**,**c**) HRTEM of point A and point B; (**d**–**f**) elemental distribution of C, Ti, and Si.

To elucidate the phase distribution within the interlayer of the joint, transmission electron microscopy was employed to examine the SiC joint annealed at 1300 °C for 20 min. The TEM observations revealed a microstructure similar to that of zone 1 in Figure 3c, where distinct Ti + C-rich and Si-rich regions were identified based on elemental mapping and morphological analysis, as illustrated in Figure 6a,d–f. The white regions correspond to the Ti + C-rich areas, while the black regions represent the Si-rich areas. Combining EDS, XRD, and TEM elemental mapping, it was confirmed that the white Ti + C-rich regions consist of TiC and TSC phases, whereas the black Si-rich regions are composed of the Ti_5_Si_3_ phase. As shown in Figure 6b,c, the characteristics of the crystal structure (SAED patterns and the set of lattice fringe spacings) corroborated the presence of the TSC phase at point A within the white region and the Ti_5_Si_3_ phase at point B within the black region. During the joining process, the TiC generated by the first reaction blocked most of the Si at both sides of the interlayer and at the matrix interface. With the extension of the holding time, the retained Si reacted with the remaining Ti and TiC to generate Ti_5_Si_3_ and TSC. Therefore, during the joining process of a 20 μm Ti foil and SiC ceramics at 1300 °C, the reaction process of the main phase components in the interlayer with different holding times can be determined as follows: Ti → Ti + TiC → TiC + TSC → TiC + TSC + Ti_5_Si_3_.

### 3.2. Microstructure and Phase Composition of SiC-Ti-SiC Joints Under Different Joining Temperatures

**Figure 7:**
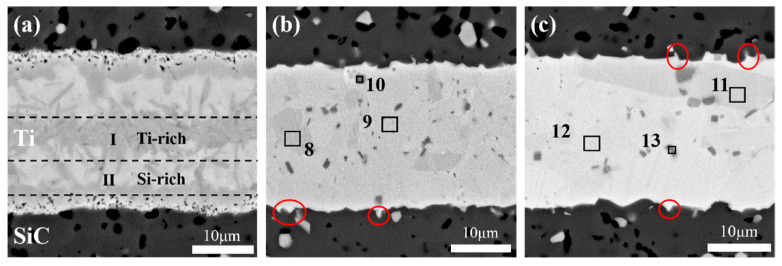
SEM images of the polished cross-sections of joints at different joining temperatures: (**a**) 1300 °C; (**b**) 1400 °C; (**c**) 1500 °C.

The influence of the joining temperature on the microstructure and mechanical properties of SiC joints was further investigated. Figure 7a–c present the polished cross-sectional microstructures of SiC ceramics joined with a 20 μm Ti foil at different joining temperatures (1300 °C, 1400 °C, and 1500 °C) under a holding time of 10 min. At a joining temperature of 1300 °C, insufficient liquefaction of the Ti interlayer limited diffusion and densification, resulting in a lower shrinkage rate. As shown in Figure 7a, this defect led to the formation of numerous pores on both sides of the interlayer. At this temperature, the interlayer exhibited an inhomogeneous microstructure with incomplete interfacial reactions, primarily divided into two distinct regions based on backscattered electron contrast: Region I (Ti-rich) and Region II (Ti-Si-rich).

When the joining temperature was increased to 1400 °C and 1500 °C (Figure 7b,c), significant microstructural evolution was observed. The reaction between Ti and SiC became more complete, and no distinct elemental diffusion or reaction layers were observed in the interlayer. Instead, blocky phases and finely dispersed particulate phases were present, indicating improved densification and uniformity of the interlayer microstructure. This suggests that higher joining temperatures effectively promote diffusion reactions between Ti and SiC. To further investigate the elemental composition of the phases in the interlayer at higher joining temperatures, EDS analysis was conducted on points 8–13 in Figure 7b,c. As shown in Table 2, the blocky phases (points 8 and 11) and the reacted interlayer (points 9 and 12) exhibited similar compositions, primarily consisting of TSC and TiC phases. This indicates that the interlayer of SiC joints joined at 1400 °C and 1500 °C had a relatively uniform phase distribution, which contributes to consistent mechanical properties across the joint and enhances overall strength. The finely dispersed particulate phases (points 10 and 13) were predominantly composed of Ti. Given the high joining temperatures, it is unlikely that unreacted or residual Ti remained in the interlayer, suggesting that these phases may have precipitated or formed during the joining process. Notably, regardless of whether the joining temperature was 1300 °C or higher, the interlayer remained continuous and tightly bonded, with no visible transverse or micro-cracks at the interface.

**Figure 8:**
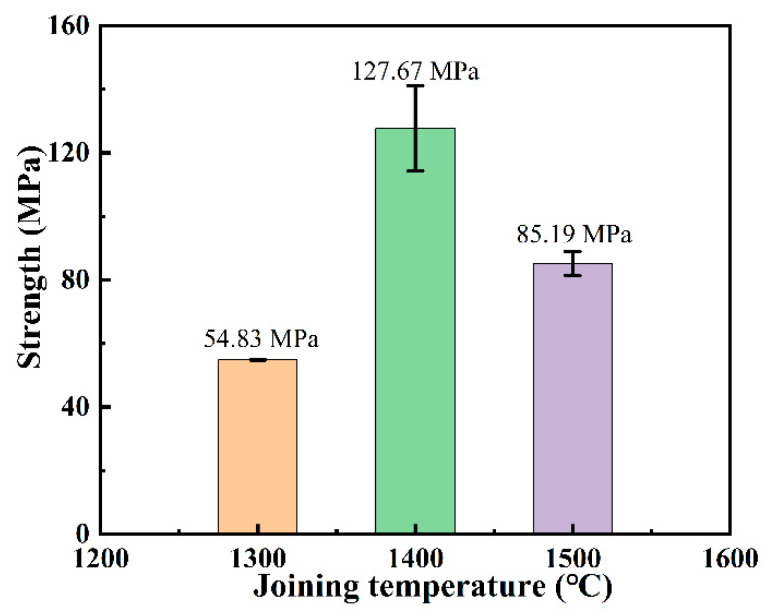
Shear strength of joints at different joining temperatures.

Figure 8 shows the shear strength of joints joined at different temperatures under a holding time of 10 min. The shear strength initially increased and then decreased with increasing joining temperature, measuring 54.83 ± 0.05 MPa at 1300 °C, 127.67 ± 13.41 MPa at 1400 °C, and 85.19 ± 3.76 MPa at 1500 °C, respectively. The lower shear strength at 1300 °C is attributed to the lack of densification in the interlayer, resulting in poor bonding strength. As shown in Figure 7b,c, the interlayer microstructure of joints joined at 1400 °C and 1500 °C exhibited typical densification and uniform phase distribution. The finely dispersed particulate phases also contributed to dispersion strengthening, enhancing joint strength. On the other hand, the increased thermal motion of Ti atoms at high temperatures improved the softness and ductility of the Ti interlayer. During the SPS joining process, the Ti interlayer gradually penetrated into the pores or micro-cracks on the SiC ceramic surface, forming a “nail-like” morphology embedded in the SiC matrix, as highlighted by the red circles in Figure 7b,c. This phenomenon, referred to as the “nail effect,” was also observed by J. Wang et al. [42] in C/C composite joints using a Ti-Si-SiC-C filler as the interlayer. The nail-like morphology creates a mechanical interlock between the interlayer and the SiC matrix, improving fracture toughness and shear strength at the bonding interface. However, in joints joined at 1500 °C, larger blocky phases were observed near the matrix interface, which could act as stress concentration zones during loading, leading to a reduction in shear strength at this temperature.

**Figure 9:**
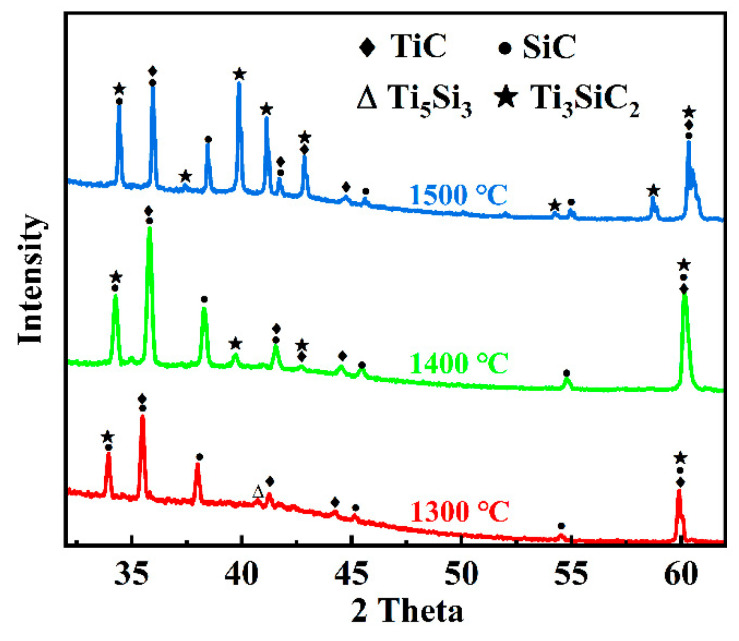
XRD patterns of the joints at different joining temperatures.

For SiC joints prepared at 1300 °C, the primary reaction phases in the interlayer were identified as Ti_5_Si_3_, TiC, and TSC. Figure 9 presents the XRD patterns of joints joined at different temperatures. The results show that the Ti_5_Si_3_ phase was nearly completely consumed at 1400 °C, leaving only TSC and TiC phases in the interlayer. Although excessive joining temperatures can lead to the decomposition of TSC [43], the short holding time prevented complete decomposition, and TSC and TiC phases were still detected in the interlayer even at 1500 °C. These findings are consistent with the earlier discussion, further confirming that higher temperatures promote more complete reactions between Ti and SiC, forming more TSC and TiC phases. The possible reactions are as follows [41,44]:

TiC + Ti_5_Si_3_ + Si → TSC (7)

TSC → Si(g) + TiC (8)

**Figure 10:**
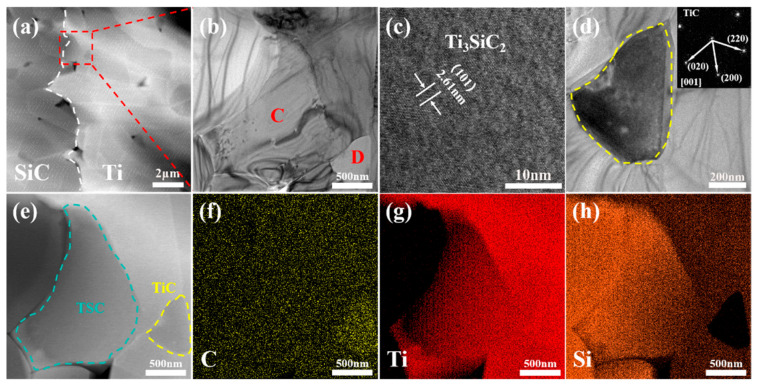
(**a**,**b**) TEM image of the joint at 1500 °C with a 20 μm Ti foil; (**c**) is HRTEM of the mark C in (**b**); (**d**) is a magnified TEM image and SAED of the marked D in (**b**); (**e**) STEM image of (**b**) and element mapping of same area: the elemental distribution of (**f**) C, (**g**) Ti, (**h**) Si.

To further investigate the phase distribution in the intermediate layer at high bonding temperatures, the microstructure of the joint bonded at 1500 °C with a 20 μm Ti foil for 10 min was examined using transmission electron microscope (TEM, Themis Z (3.2), Thermo Fisher Scientific, Brno, Czech Republic). By analyzing the crystal lattice fringe spacing and electron diffraction patterns (Figure 10c,d), the phases marked as C and D in Figure 10b are confirmed to be TSC and TiC, respectively. This result is consistent with the elemental distribution in the corresponding regions, further validating the accuracy of the phase identification.

Additionally, the TEM images reveal distinct grain boundaries and phase boundaries in the joint, which may influence the mechanical properties and thermal stability of the material. In particular, the TSC phase shown in Figure 10c exhibits clear crystal orientation and lattice fringes, indicating high crystallinity, which is highly beneficial for the overall performance of the material.

In summary, at higher bonding temperatures, the reaction between Ti and SiC becomes more thorough, leading to a significant increase in the content of TSC and TiC in the intermediate layer and the formation of stable interfacial bonding. This contributes to an overall improvement in the joint strength.

### 3.3. Microstructure and Phase Composition of SiC-Ti-SiC Joints Under Different Thicknesses of Ti Foils

**Figure 11:**
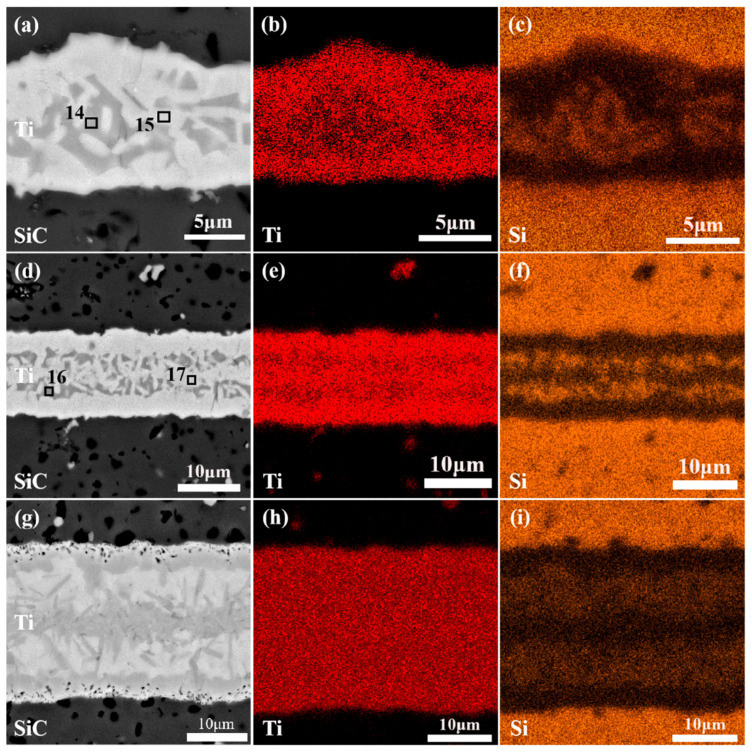
Microstructure and element distribution maps of SiC joints with different Ti foil thicknesses: (**a**) 5 μm; (**b**) distribution of Ti; (**c**) distribution of Si; (**d**) 10 μm; (**e**) distribution of Ti; (**f**) distribution of Si; (**g**) 20 μm; (**h**) distribution of Ti; (**i**) distribution of Si.

To investigate the influence of interlayer thickness on the microstructure and properties of joints, SiC ceramics were joined using Ti foils of different thicknesses. Figure 11 shows the microstructure and elemental surface distribution of SiC joints prepared at a bonding temperature of 1300 °C and a holding time of 10 min. When the initial thicknesses of the Ti foils were 5 μm, 10 μm, and 20 μm, the thicknesses of the interlayers after joining increased to 8.68 μm, 14.04 μm, and 26.57 μm, respectively. This is due to the reaction between SiC and Ti at the bonding temperature, forming TiC and Si phases. Driven by chemical potential, atomic-level mixing of Si and Ti occurred, leading to significant expansion of the Ti interlayer after joining. Comparative analysis of Figure 11a,d,g reveals that the microstructure of the SiC joints changes significantly with variations in Ti foil thickness. When 5 μm and 10 μm Ti foils were used as interlayers, in the central region of the interlayer, granular phase structures of a dark gray phase with a deeper contrast have emerged, with clear interfacial transition zones on both sides. Notably, the granular phases in the 5 μm interlayer were relatively smaller in size and uniformly distributed, while those in the 10 μm interlayer were larger in size and unevenly distributed, showing a tendency for the granular phases to become segmented. In contrast, the 20 μm Ti interlayer exhibited a more complex dendritic phase structure. To further clarify the elemental distribution, EDS mapping was used to visualize the distribution of elements within the joints. The distribution of Si, which tends to diffuse into the interlayer, is shown in Figure 11c,f,i. The results indicate that the dark gray granular phases in the thinner Ti interlayers were identified as Si-rich phases, and Si-rich phases were also detected in the 20 μm Ti interlayer, primarily distributed near the edges of the interlayer. From a diffusion kinetics perspective, during the SPS heating process, the local interfacial temperature of the joint generally exceeds the bonding temperature, allowing some rapidly softened Si to reach the Ti interlayer before the formation of TiC barriers. However, the diffusion of Si atoms in the Ti matrix primarily relies on a thermally activated process, with the diffusion rate influenced by temperature and time. Additionally, the increase in Ti interlayer thickness means that Si atoms must travel a longer distance, and the extended diffusion path increases diffusion resistance. Therefore, in the thinner interlayers (5 μm and 10 μm), due to the limited interlayer thickness, Ti atoms and Si atoms from SiC can fully diffuse within a short time, leading to the formation of a large number of Si-rich phases in the central region. Under the same bonding temperature and holding time, as the Ti foil thickness increases to 20 μm, the diffusion of Ti and Si atoms in the edge regions of the interlayer is relatively slower and more uniform, resulting in the gradual formation and dispersed distribution of Si-rich phases near the edges. As diffusion proceeds, the elemental concentration gradient within the interlayer gradually becomes more uniform, forming a relatively homogeneous microstructure.

**Figure 12:**
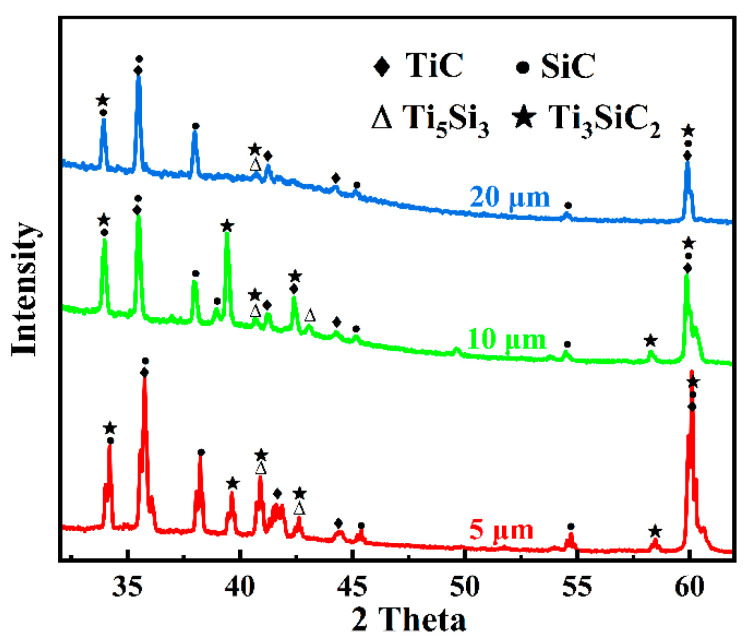
XRD of joints with different Ti foil thicknesses.

To determine the elemental composition of the Si-rich phases and other regions, point scanning was performed at selected points in Figure 11a,d. Table 3 provides the chemical composition percentages at these points. The results show that the atomic ratios of Ti to Si at points 14 and 16, which represent the granular phases, are close to 5:3, strongly suggesting the presence of Ti_5_Si_3_. At points 15 and 17, the C content is significantly higher, while the Si content is lower, indicating the possible existence of Ti-Si-C ternary compounds, such as TSC, as well as free TiC phases. XRD analysis further confirms this speculation. As shown in Figure 12, in addition to the SiC matrix, characteristic diffraction peaks of TSC, TiC, and Ti_5_Si_3_ are observed in SiC joints with different interlayer thicknesses. Generally, the intensity of diffraction peaks reflects the relative content of the corresponding phases. By comparing the diffraction peak intensities, it is evident that as the Ti interlayer thickness increases from 5 μm to 20 μm, the content of Ti_5_Si_3_ and TSC decreases significantly. This indicates that thicker Ti interlayers may slow down the reaction rates between Si and Ti to form compounds such as Ti_5_Si_3_ and TSC. These reactions typically depend on the diffusion of Si atoms and their contact with Ti atoms. This is mainly because thicker Ti interlayers may hinder the diffusion of Si atoms, and the reaction kinetics between Si and Ti atoms are also influenced by the thickness of the Ti interlayer. In summary, at a bonding temperature of 1300 °C, when Ti foils of different thicknesses are used as interlayers to join SiC, the main reaction products in the interlayer remain TSC, TiC, and Ti_5_Si_3_ phases.

**Figure 13:**
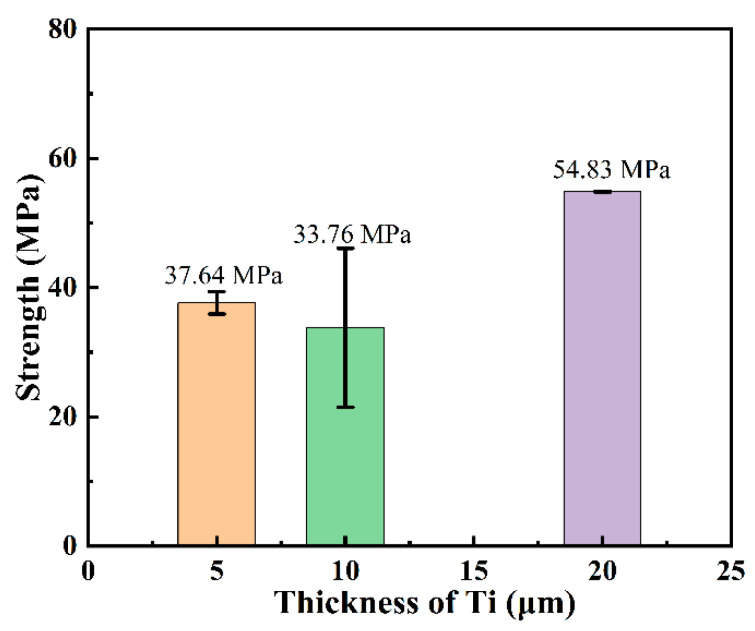
Shear strength of joints with different Ti foil thicknesses.

Figure 13 shows the shear strength test results of SiC joints joined using Ti foils of different thicknesses. The shear strength was 37.64 ± 1.73 MPa for 5 μm, 33.76 ± 12.31 MPa for 10 μm, and 54.83 ± 0.05 MPa for 20 μm, respectively. The results indicate that when Ti foils with thicknesses of 5 μm and 10 μm are used as interlayers, the shear strength of the joints is significantly lower than that of joints with a 20 μm Ti interlayer. From a microstructural perspective, the central region of the thinner Ti interlayers contains densely distributed Ti_5_Si_3_ phases, indicating the aggregation of a large number of brittle phases primarily in the center of the interlayer. These aggregated phases further exacerbate stress concentration within the joint, making it easier for cracks to initiate and propagate under shear loading, leading to premature failure. Additionally, the thinner interlayer results in a relatively shorter and more concentrated crack propagation path, causing the joint to undergo rapid brittle fracture and exhibit lower shear strength. In contrast, in the thicker Ti interlayer, these brittle phases are more dispersed, resulting in a more uniform stress distribution within the joint and greater distances between the dispersed phases. This increases the energy barrier for crack propagation and extends the crack path, enabling the 20 μm Ti interlayer joint to withstand higher shear loads and exhibit greater shear strength during shear testing.

**Figure 14:**
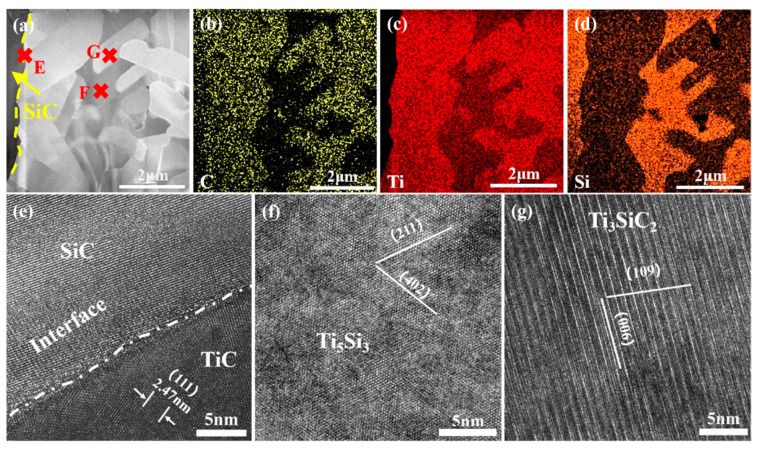
(**a**) TEM image of the sample joined at 1300 °C with a 5 μm Ti; (**b**–**d**) elemental distributions of C, Ti, and Si; (**e**–**g**) HRTEM of mark E, F, and G in (**a**), respectively.

The microstructure of a joint with a 5 μm Ti interlayer was analyzed using transmission electron microscope (TEM, Themis Z (3.2), Thermo Fisher Scientific, Brno, Czech Republic). Figure 14 presents the high-angle annular dark-field (HAADF) image of the joint interface connected with a 5 μm Ti foil, along with the corresponding elemental distributions of C, Ti, and Si. Consistent with the observations from scanning electron microscopy, the Si-rich phase was predominantly concentrated in the central region of the interlayer, surrounded by the Ti-rich phase. Further analysis of the marked regions E, F, and G was conducted using high-resolution transmission electron microscopy (HR-TEM), as shown in Figure 14e,f. At the joint interface, a nanoscale TiC phase was observed, with lattice fringe spacings closely matching the theoretical value of TiC (0.249 nm for the (111) plane), confirming the occurrence of an interfacial reaction between Ti and SiC, leading to the formation of TiC compounds. The Si-rich phase (marked F) and Ti-rich phase (marked G) identified by energy-dispersive X-ray spectroscopy mapping were further characterized by HR-TEM as the Ti_5_Si_3_ metallic phase and the MAX phase TSC, respectively. Notably, the lattice fringes in each phase region exhibited good continuity, with no significant amorphous layers or defect aggregation observed, indicating high-quality joint formation under these conditions. The lower joint strength observed when using a thinner Ti interlayer to connect SiC is primarily attributed to the aggregation of the Si-rich Ti_5_Si_3_ phase in the central region of the interlayer, which leads to stress concentration during shear loading, resulting in low-strength fracture.

## 4. Conclusions

This study systematically investigated the diffusion bonding of SiC ceramics using Ti interlayers via the SPS process, with key findings summarized as follows:

### 4.1. Effect of Holding Time

The SiC joints exhibited similar microstructural characteristics across different holding times, with the interlayer consistently composed of TSC, TiC, and Ti_5_Si_3_ phases. The shear strength analysis revealed a positive correlation with holding duration, peaking at the 20 min condition. However, this extended holding time induced significant phase segregation within the interlayer, as confirmed by microstructural characterization, which directly compromised joint reliability through increased mechanical instability. Significantly, the 10 min holding condition demonstrated optimal performance balance, producing joints with superior phase homogeneity and enhanced strength reproducibility.

### 4.2. Effect of Joining Temperature

As the joining temperature increased, the interfacial reaction between the Ti interlayer and SiC substrate became more complete, resulting in gradual consumption of the Ti_5_Si_3_ phase and significant enrichment of TSC and TiC phases in the interlayer. Microstructural evolution analysis revealed a unique “nail effect”—mechanical interlocking at the interface formed by Ti-based phases penetrating substrate defects. This mechanistic transition contributed to a remarkable improvement in shear strength, with the 1400 °C process achieving peak strength (127.67 MPa) through optimal phase distribution. However, when the temperature was further increased to 1500 °C, abnormal coarsening of TiC phases occurred, creating stress concentration effects that reduced the strength to 85.19 MPa (±3.76 MPa).

### 4.3. Effect of Ti Foil Thickness

Systematic investigation of Ti interlayers with varying thicknesses revealed that while all joints maintained the characteristic TSC-TiC-Ti_5_Si_3_ phase composition, significant microstructural differences were observed. Thin interlayers (5 and 10 μm) induced pronounced segregation of Si-rich phases in the central region of the interlayer, creating localized stress concentrations that substantially reduced shear strength. In contrast, the 20 μm interlayer demonstrated homogeneous phase distribution, enabling effective stress redistribution and achieving superior shear strength of 54.83 MPa.

## Figures and Tables

**Table 1:** EDS of each point marked in Figure 3a–c.

Point	Composition in Atomic %			Probable Phase
	Ti	Si	C	
1	74.93	13.08	11.99	Ti_3_SiC_2_, TiC
2	69.59	23.64	6.77	Ti_5_Si_3_
3	73.75	12.32	13.92	Ti_3_SiC_2_, TiC
4	69.38	23.79	6.74	Ti_5_Si_3_
5	59.81	21.00	19.19	Ti_3_SiC_2_, TiC
6	68.32	24.24	7.44	Ti_5_Si_3_
7	55.72	36.86	7.42	Ti_5_Si_3_

**Table 2:** EDS of each point marked in Figure 7b,c.

Point	Composition in Atomic %			Probable Phase
	Ti	Si	C	
8	71.48	13.29	15.23	Ti_3_SiC_2_, TiC
9	71.60	13.25	15.15	Ti_3_SiC_2_, TiC
10	96.22	3.78	0.00	Ti
11	72.27	12.78	14.95	Ti_3_SiC_2_, TiC
12	71.02	13.09	15.90	Ti_3_SiC_2_, TiC
13	94.23	5.05	0.72	Ti

**Table 3:** EDS of each point marked in Figure 11a,d.

Point	Composition in Atomic %			Probable Phase
	Ti	Si	C	
14	55.72	36.86	7.42	Ti_5_Si_3_
15	59.80	21.00	19.19	Ti_3_SiC_2_, TiC
16	55.65	36.78	7.56	Ti_5_Si_3_
17	66.98	16.02	17.00	Ti_3_SiC_2_, TiC

## Data Availability

The original contributions presented in this study are included in the article. Further inquiries can be directed to the corresponding authors.
